# Supplementary material for: Stabilizing dual-phased perovskite towards high performance photovoltaics with enhanced batch stability and consistency
Source: Nat Commun. 2025 Sep 30;16:8681. doi: 10.1038/s41467-025-63776-6 (PMC12484941; doi:10.1038/s41467-025-63776-6)
Supplement: Supplementary file 1 — supplementary information [file 41467_2025_63776_MOESM1_ESM.pdf]

Stabilizing Dual-Phased Perovskite Towards High Performance Photovoltaics with Enhanced Batch Stability and Consistency

Guihua Zhang,#[1-3] Deng Wang,#[1][4] Bowei Li,#[5] Qing Lian,#[1]* Xinyi Zou,[6] Dongyang Li,[1] Qiming Yin,[1] Guojun Mi,[1] Jie Li,[1] Kui Feng,[1] Abbas Amini,[7] Alex K.-Y. Jen,[4] Xugang Guo,[1] Baomin Xu, [1]* and Chun Cheng [1-3]*

[1]G. Zhang, D. Wang, Q. Lian, D. Li, Q. Yin, G. Mi, J. Li, K. Feng, X. Guo, B. Xu, C. Cheng
Department of Materials Science and Engineering, Southern University of Science and Technology, Shenzhen 518055, China
Emails: lianq@sustech.edu.cn, xubm@sustech.edu.cn, chengc@sustech.edu.cn

[2]G. Zhang, C. Cheng
Guangdong Provincial Key Laboratory of Energy Materials for Electric Power, Southern University of Science and Technology, Shenzhen, 518055, China

[3]G. Zhang, C. Cheng
SUSTech Energy Institute for Carbon Neutrality, Southern University of Science and Technology, Shenzhen, 518055, China

[4]D. Wang, A. K.-Y. Jen
Department of Materials Science and Engineering, City University of Hong Kong, Tat Chee Ave, 999077 Kowloon, China

[5]B. Li
Future Photovoltaic Research Center, Global Institute of Future Technology, Shanghai Jiao Tong University, Shanghai 200240, China

[6]X. Zou

Department of Chemistry, Southern University of Science and Technology, Shenzhen 518055, China.

[7]A. Amini
Urban Transformations Research Centre, Western Sydney University, Penrith 2751, NSW, Australia.

#These authors contributed equally to this work.

Supplementary Figures

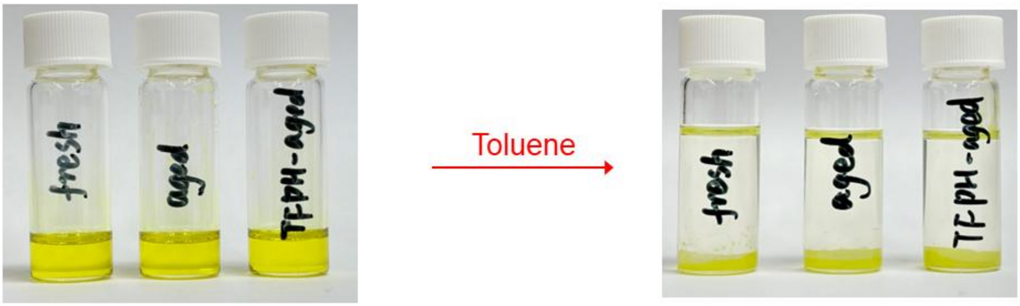

Figure S1. The observation by naked eyes on different perovskite solutions and their I_2 extracted solutions by toluene.

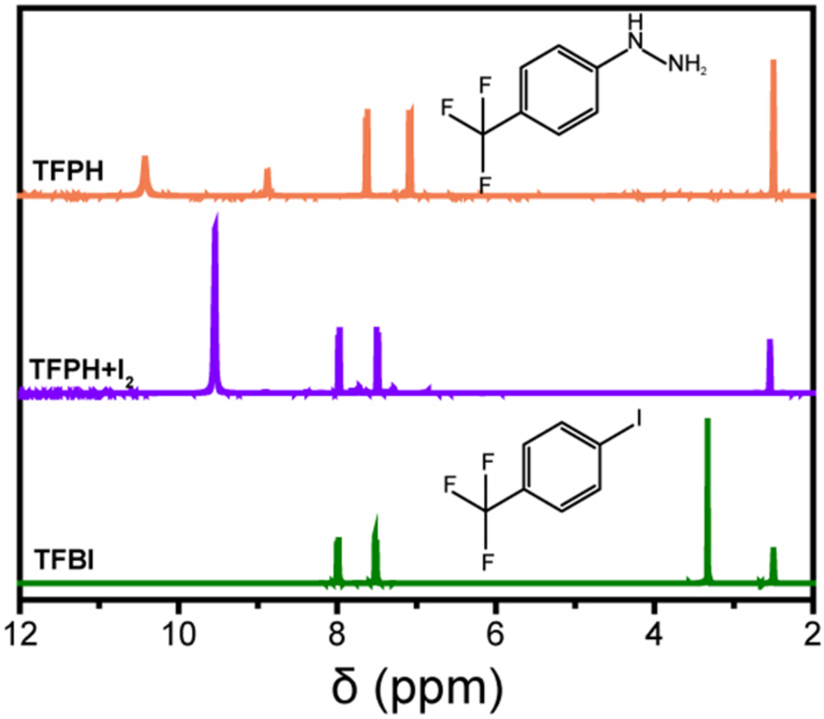

Figure S2. ^1H NMR spectra of TFPH, TFPH+I_2 and TFBI in DMSO-d_6.

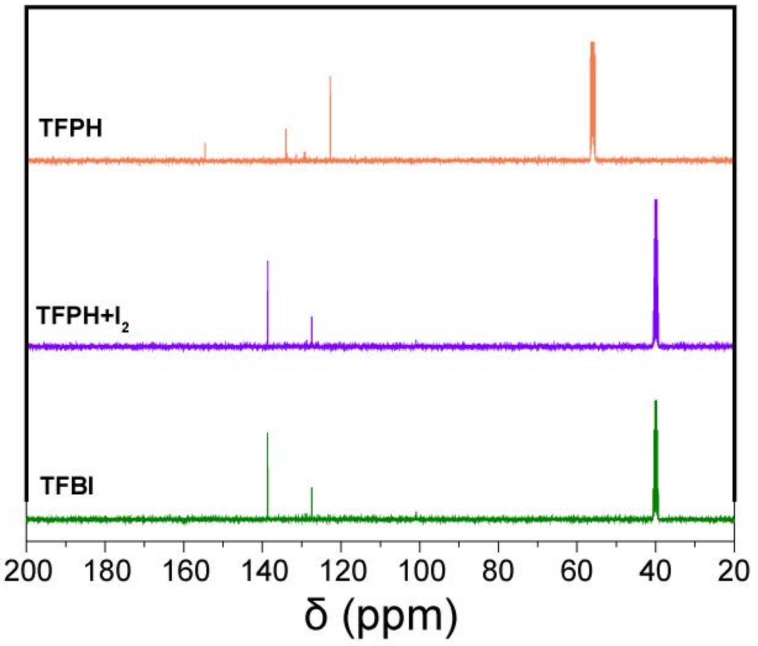

Figure S3. ^{13}C NMR spectra of TFPH, TFPH+I_2 and TFBI in DMSO-d_6

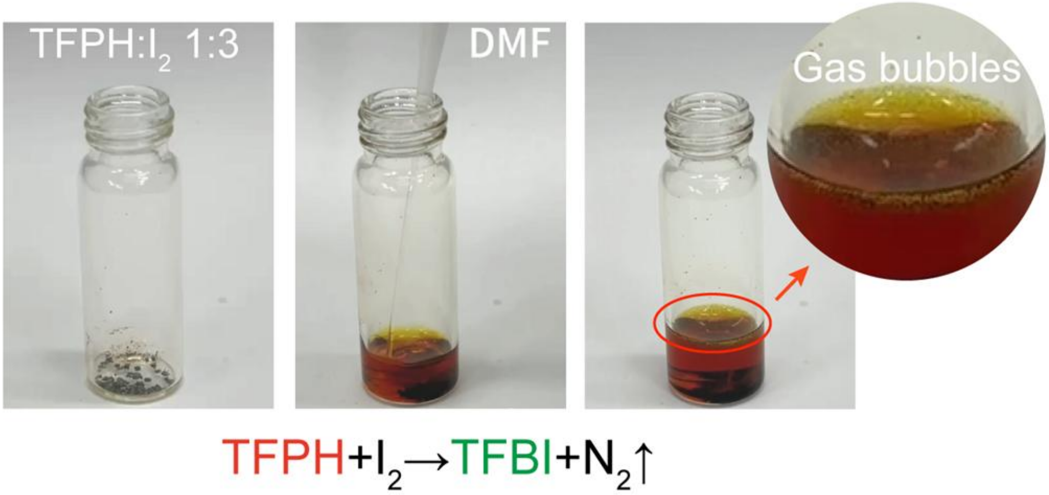

Figure S4. The production of gas during mixing TFPH、I_2 and DMF.

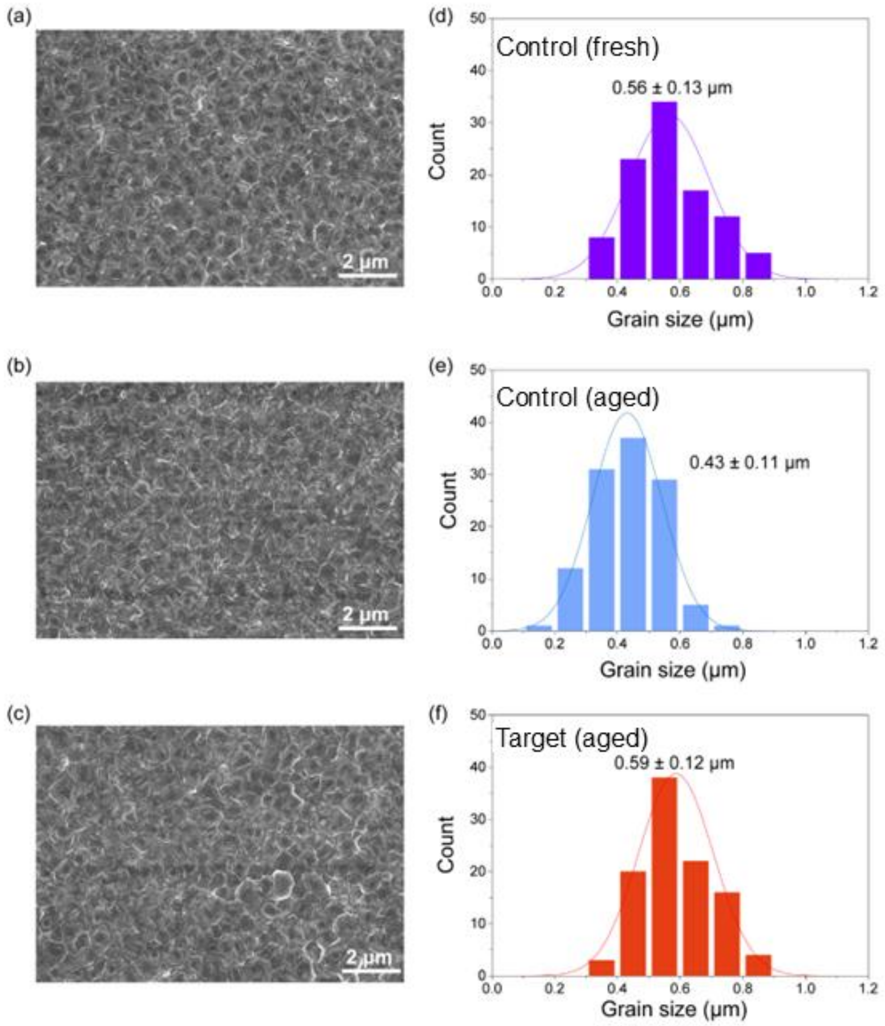

Figure S5. Morphology of the perovskite films. a-c) Top-view SEM images of the perovskite films. d-f) Statistical distributions of the grain size.

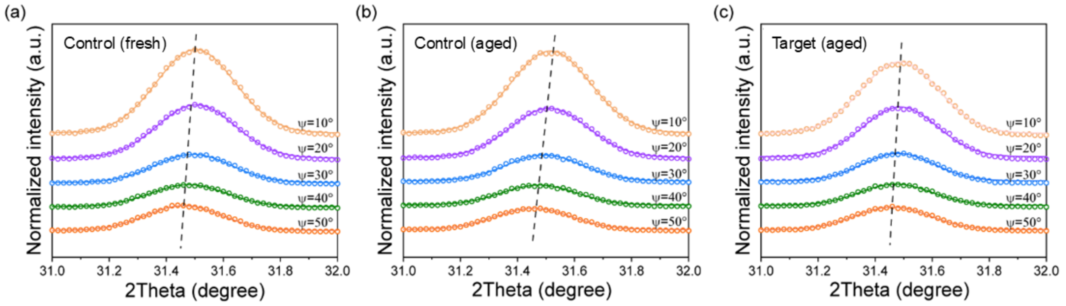

Figure S6. GIXRD spectra of the perovskite films at different tilt angles (ψ). a) Control (fresh), b) Control (aged), c) Target (aged).

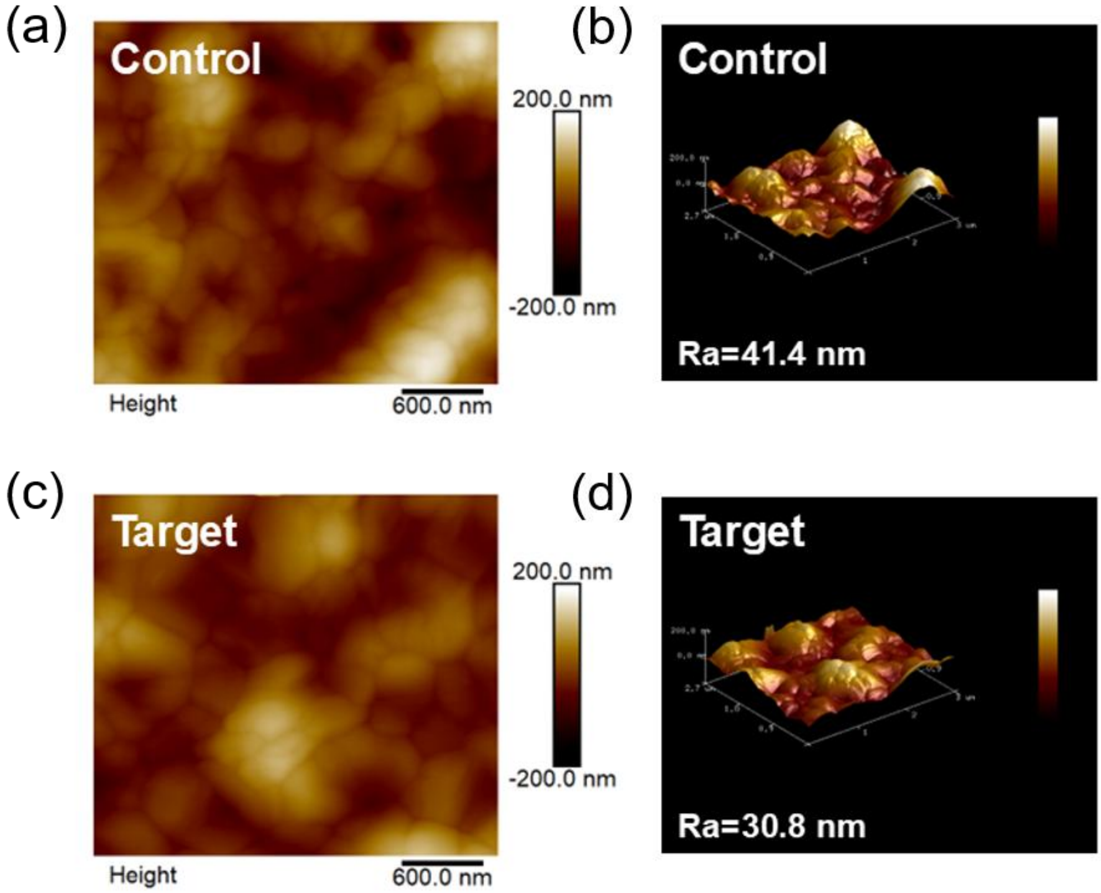

Figure S7. AFM images of the perovskite films. a) and c) show the surface height profiles of control and target perovskite films, c) and d) present their 3D surface topography and corresponding roughness.

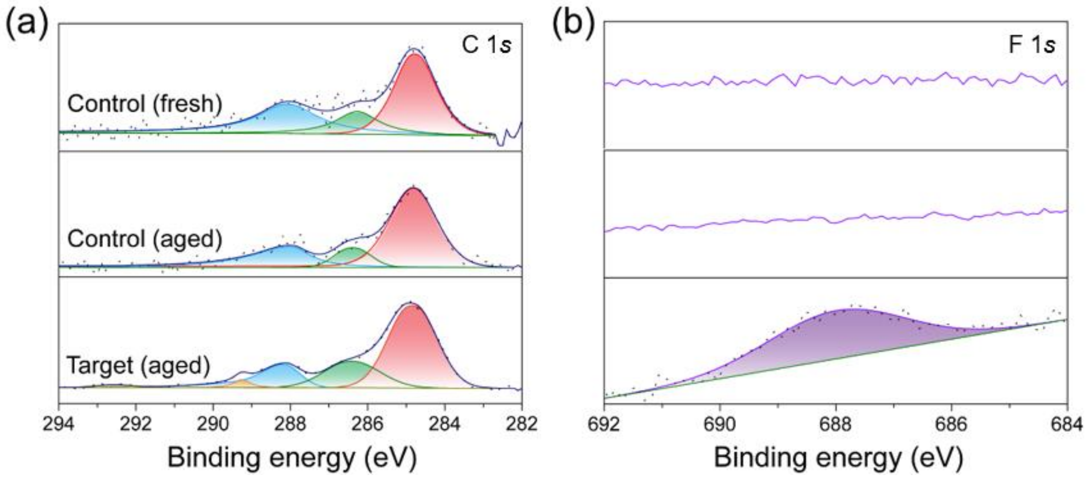

Figure S8. XPS core-level spectra of a) C 1*s* and b) F 1*s* collected from the top surfaces of perovskite films for Control (fresh), Control (aged), and Target (aged) samples.

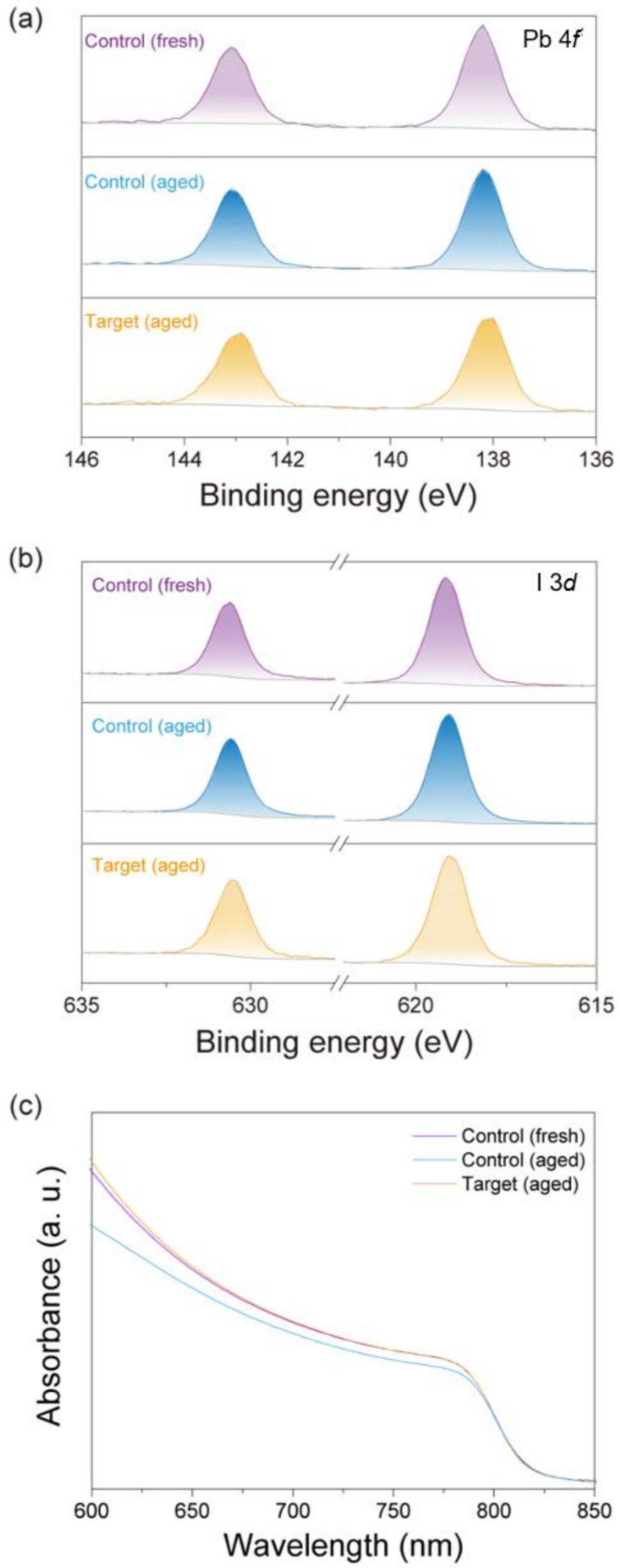

Figure S9. Optical properties of the perovskite film. High-resolution XPS spectra of a) Pb 4*f* and b) I 3*d*. c) UV-Vis spectra. .

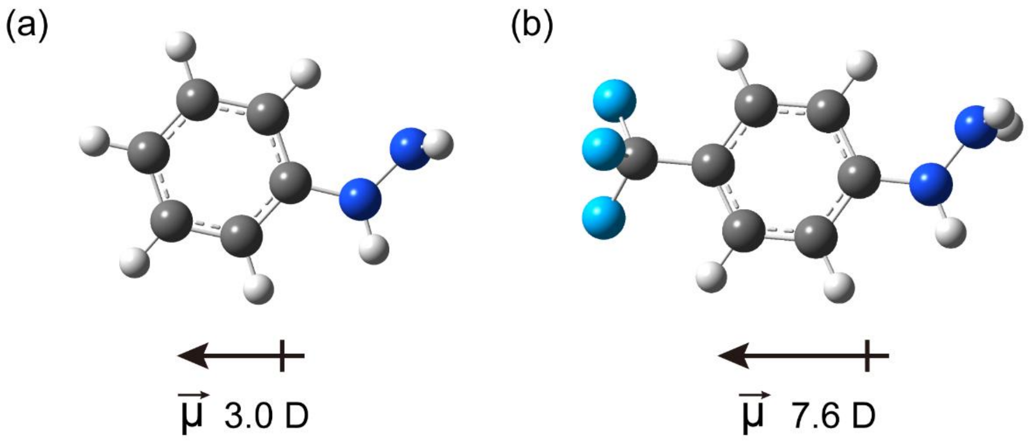

Figure S10. The dipole moment of a) phenylhydrazine (PH) and b) 4-(trifluoromethyl) phenyl hydrazine (TFPH). The dipole moment of TFPH is 2.5 times greater than that of PH.

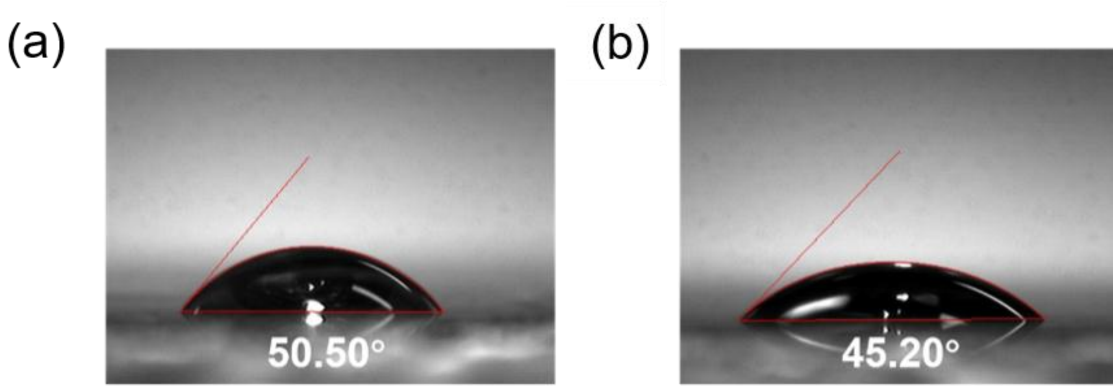

Figure S11. Contact angle of perovskite precursor solutions on SAMs modified ITO surface: a) the control (perovskite precursor without TFPH) b) the target (perovskite precursor with TFPH).

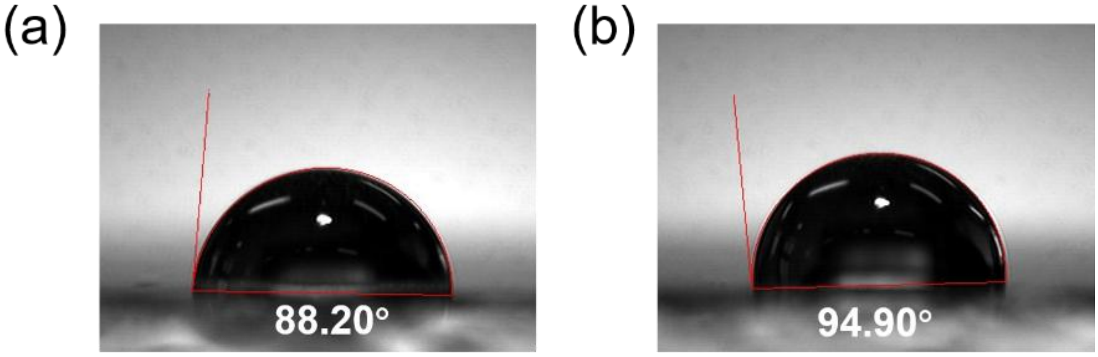

Figure S12. Contact angle of water on perovskite films: a) the control without TFPH b) the target with TFPH.

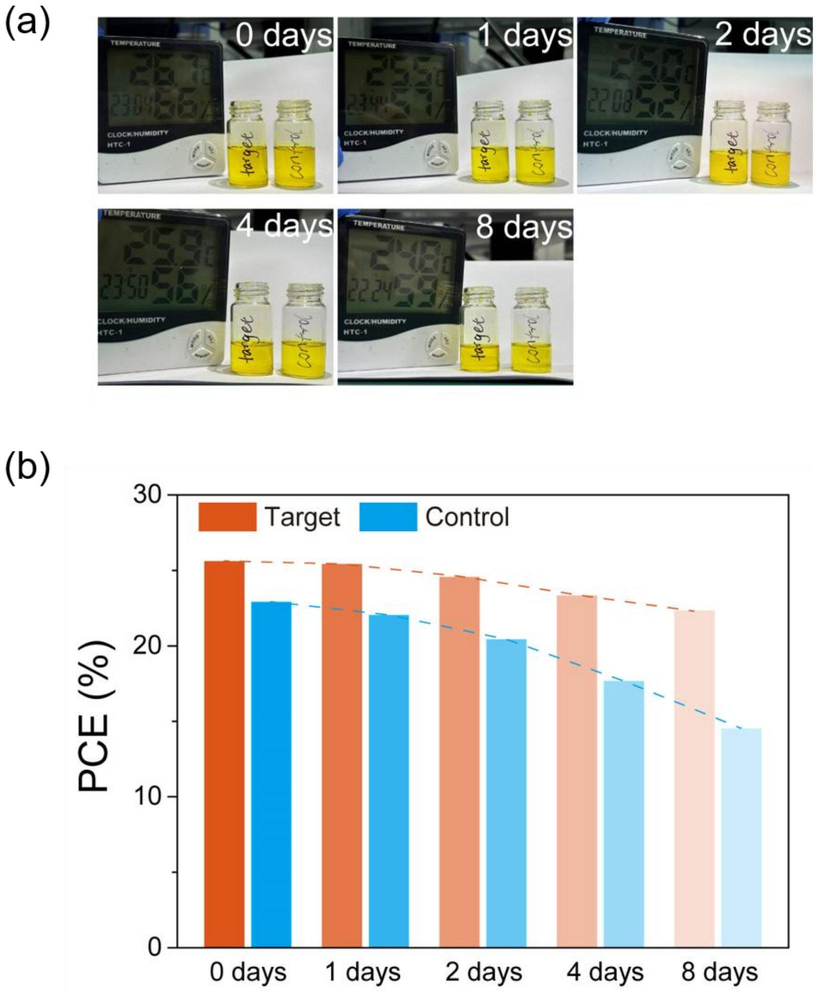

Figure S13. a) The precursor solution with and without TFPH doping aged in air, the temperature and relative humidity. b) Statistic data of perovskite solution stored in air, at various ageing times.

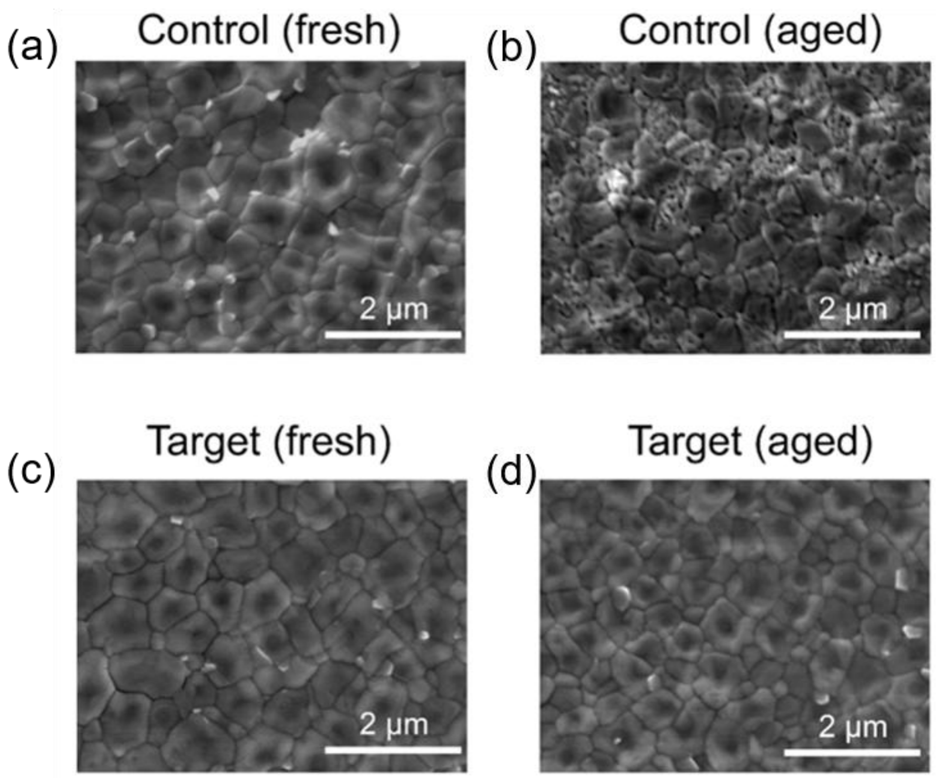

Figure S14. SEM shows the morphology of a)-b) the Control perovskite films before and after 200 hours of light soaking, and c)-d) the Target perovskite films before and after 200 hours of light soaking.

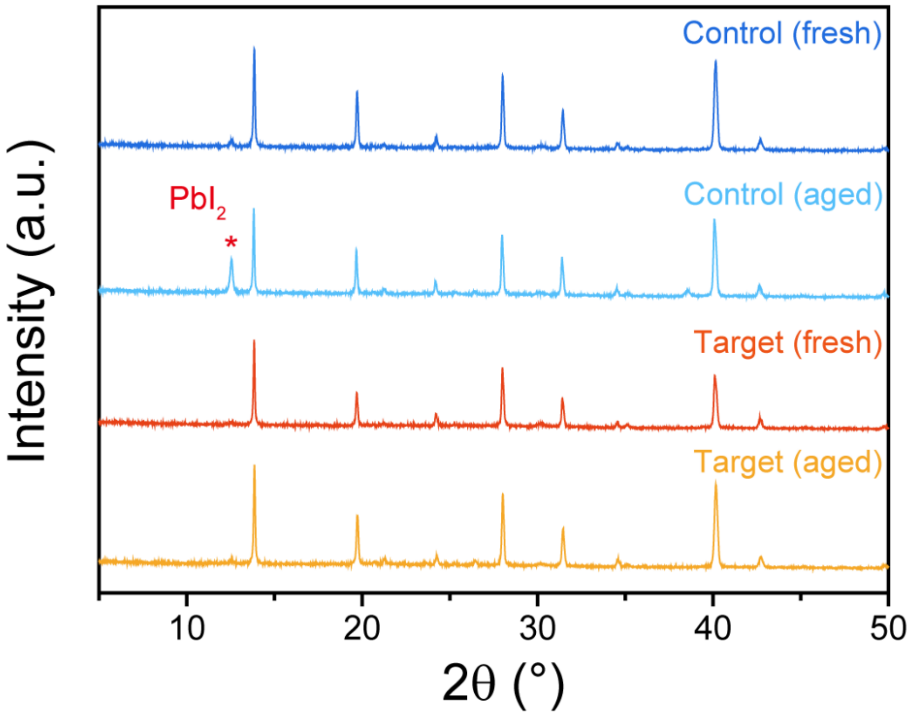

Figure S15. XRD shows the structure of the control and target perovskite films before and after 200 hours of light soaking.

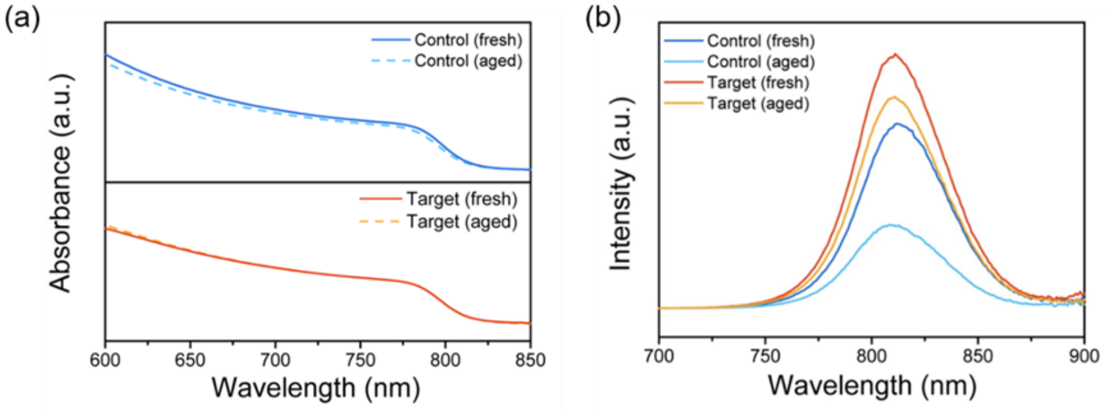

Figure S16. Optical properties of the control and target perovskite films before and after 200 hours of light soaking. a) UV-Vis spectra. b) PL.

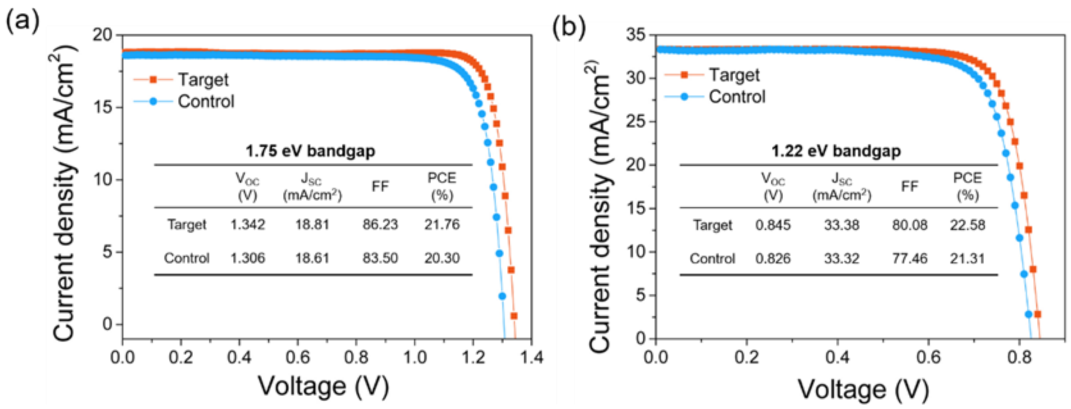

Figure S17. *J-V* curves of PSCs based on a) wide-bandgap and b) narrow-bandgap perovskite. Note that the wide-bandgap PSCs hold a structure FTO/SAMs/perovskite (1.75 eV)/C_{60}/BCP/Cu whilst the narrow-bandgap PSCs adopt a structure of FTO/PEDOT:PSS/perovskite (1.25 eV)/C_{60}/BCP/Cu.

Supplementary Tables

Table S1. Integral intensity of peak area a function of I_2/TFPH ratios. The data were obtained from ^{1}H NMR.

I_2/TFPH ratio	TFPH %	TFBD %	TFBI %
0	100	0	0
0.1	96.2	1.9	1.9
0.5	83.4	8.3	8.3
1	69.0	13.8	17.2
2	42.4	20.3	37.3
3	17.2	25.0	57.8
4	0	20.0	80.0
5	0	13.0	87.0
6	0	9.1	90.9
8	0	7.4	92.6
10	0	6.5	93.5

Table S2. Comparison of TFPH and other addtives in film characteristics and PCE.

Additive	Grain size / nm	XRD FWHM /°	PCE / %	References
1-(3-(trifluoromethyl)phenyl) thiourea	319.4	0.233	24.7	[1]
saccharin sodium	NA	0.090	24.8	[2]
trifluorophenylacetic acid	1250	0.95	24.56	[3]
phenylhydrazine-4-sulfonic acid	3000	0.07	25.10	[4]
sulfaguanidine	124	NA	24.34	[5]
4-(trifluoromethyl) phenylhydrazine	590	0.084	26.0	This work

Table S3. Trap-filled limit Voltages (V_{TFL}) and the trap density (n_{trap}) obtained from the dark *I-V* curves of the hole-only devices.

Device	V_{TFL} (V)	n_{trap} (cm^{-3})
Control (fresh)	0.091	1.03×10^{15}
Control (aged)	0.096	1.09×10^{15}
Target (aged)	0.081	0.92×10^{15}

Table S4. TRPL fitting of perovskite films with or without TFPH. Note that the films were coated on ITO/SAMs substrate.

Device	τ_1	A_1	τ_2	A_2	$\tau_{average}$
Control (fresh)	157.9	4.77	2485.9	95.23	2478.6
Control (aged)	375.7	34.6	1275.3	65.41	1154.1
Target (aged)	45.9	0.77	3821.7	99.23	3821.3

Table S5. Photovoltaic parameters of PSCs prepared from the control solution at different aging times.

Time	Data	PCE %	FF%	V_{OC} (V)	J_{SC} (mA cm^{-2})
1	Average	22.99±0.40	81.55±0.92	1.114±0.006	25.38±0.42
	Champion	23.71	82.59	1.121	25.45
15	Average	21.39±0.74	79.39±0.95	1.117±0.003	24.37±0.51
	Champion	22.70	80.28	1.122	25.24
30	Average	19.58±1.12	77.67±2.24	1.124±0.006	23.01±0.93
	Champion	21.74	81.77	1.136	23.25
60	Average	16.82±2.07	76.06±2.98	1.118±0.009	20.68±1.61
	Champion	20.21	79.85	1.135	22.23

Table S6. Photovoltaic parameters of PSCs prepared from the target solution at different aging times.

Time	Data	PCE %	FF%	V_{OC} (V)	J_{SC} (mA cm^{-2})
1	Average	25.66±0.29	84.77±1.01	1.179±0.008	25.66±0.14
	Champion	25.95	85.70	1.186	25.53
15	Average	25.67±0.31	84.69±1.03	1.180±0.006	25.68±0.16
	Champion	25.98	85.72	1.186	25.56
30	Average	25.67±0.33	84.92±0.49	1.179±0.007	25.65±0.15
	Champion	26.00	85.35	1.185	25.71
60	Average	25.62±0.29	84.69±0.63	1.178±0.006	25.69±0.15
	Champion	25.91	85.32	1.184	25.64

Table S7. Integrated J_{SC} from EQE spectra.

Device	Integrated J_{SC} (mA cm^{-2})
Control (fresh)	24.94
Control (aged)	20.67
Target (aged)	24.93

Table S8. Photovoltaic parameters of the champion PSCs prepared from the control solution at different aging times in air.

Time	Data	PCE %	FF%	V_{OC} (V)	J_{SC} (mA cm^{-2})
0	Champion	23.32	81.90	1.131	25.19
1	Champion	22.33	80.17	1.097	25.39
2	Champion	20.71	77.64	1.069	24.94
4	Champion	18.32	71.64	1.063	24.05
8	Champion	15.77	62.66	1.053	23.89

Table S9. Photovoltaic parameters of the champion PSCs prepared from the target solution at different aging times in air.

Time	Data	PCE %	FF%	V_{OC} (V)	J_{SC} (mA cm^{-2})
0	Champion	25.83	85.42	1.167	25.91
1	Champion	25.54	85.68	1.163	25.65
2	Champion	24.91	83.37	1.156	25.84
4	Champion	23.88	82.71	1.153	25.05
8	Champion	22.51	80.90	1.118	24.89

Table S10. Summary of operational stability of high-performance inverted PSCs based on ISOS-L-3.

Device structure	Best PCE	Lifetime	Reference
ITO/Me-4PACz/Al_2O_3/ $Cs_{0.05}MA_{0.1}FA_{0.85}PbI_3$/PCBM/$C_{60}$/$SnO_x$/Ag	26.02%	$T_{96.9}$=1246 h	*Adv. Sci.* **2024**, 2407380.
ITO/NiO_x/$Cs_{0.05}FA_{0.85}MA_{0.1}PbI_3$/ PCBM/BCP/Ag	23.91% (certified)	T_{92}=500 h	*Nat. Photon.* **2022**, 16, 352–358.
ITO/2PACz/$Cs_{0.05}MA_{0.05}FA_{0.9}Pb(I_{0.95}Br_{0.05})_3$/ C_{60}/BCP/Ag	24.09% (certified)	T_{85}=1560 h	*Science* **2023**, 381,209-215.
FTO/2PACz:3-MPA/ $Cs_{0.05}MA_{0.10}FA_{0.85}PbI_3$/345FAn/$C_{60}$/BCP/Ag	25.3%	T_{95}=1075 h	*Nature* **2023**, 624, 289–294.
FTO/NiO_x/4PACz/ $Cs_{0.05}MA_{0.05}FA_{0.90}PbI_3$/$C_{60}$/BCP/Ag	26.4%	T_{96}=2000 h	*Science* **2023**, 382,810-815.
FTO/2PACz:Me-4PACz/ $Cs_{0.05}MA_{0.1}FA_{0.85}PbI_3$ /BMP/C_{60}	26.9%	T_{95}=1200 h	*Science* **2024**, 384,189-193.
ITO/PTAA/$Rb_{0.05}Cs_{0.05}MA_{0.05}FA_{0.85}Pb(I_{0.95}Br_{0.05})_3$/$C_{60}$/BCP/Cu	25.2%	T_{85}=500 h	*Nature* **2024**, 625, 516–522.
ITO/2PADCB/$FA_{0.95}Cs_{0.05}PbI_3$/C_{60}/BCP/Cu	**26.0%**	T_{92}=1830 h	**This work**

Table S11. List of raw material unit prices.

Materials	Price (USD)	Per unit
ITO glass	0.5	piece (1.5×1.5 cm^2)
FAI	15	g
PbI_2	2	g
CsI	4	g
C_{60}	167	g
BCP	470	g
Cu	1	g
TFPH	1	g
DMF	84	L
DMSO	575	L
CB	200	L

Table S12. The cost-benefit relationship enabled by the TFPH additive.

	Control	Target	Comparison
Cost per 1 mL precursor (USD)	6.3573	6.3623	+0.079 %
Cost per device (USD)	0.784168	0.784368	+0.026 %
Average PCE (%)	22.99	25.66	+11.62 %

Supplementary References

[1] Hua, G. *et al.* Thiourea Suppressing Iodide Oxidation and Passivating Perovskite Surface to Achieve High-Efficiency Stable Solar Cells. *Advanced Functional Materials* **35,** 2414423 (2024).

[2] Yang, Z. *et al.* Stabilize Perovskite Precursors and Inhibit Intermediates for High Performing Perovskite Solar Cells. *Small* **21,** 2503279 (2025).

[3] Cao, Y. *et al.* Bifunctional Trifluorophenylacetic Acid Additive for Stable and Highly Efficient Flexible Perovskite Solar Cell. *InfoMat* **5**, e12423 (2023).

[4] Liu, H. *et al.* Defect Management and Ion Infiltration Barrier Enable High-Performance Perovskite Solar Cells. *ACS Energy Letters* **9**, 2790-2799 (2024).

[5] Zhou, W. *et al.* Achieving High-Quality Perovskite Films with Guanidine-Based Additives for Efficient and Stable Methylammonium-Free Perovskite Solar Cells. *Advanced Functional Materials* **34**, 2407897 (2024).
