# Supplementary material for: Stabilizing dual-phased perovskite towards high performance photovoltaics with enhanced batch stability and consistency
Source: Nat Commun. 2025 Sep 30;16:8681. doi: 10.1038/s41467-025-63776-6 (PMC12484941; doi:10.1038/s41467-025-63776-6)
Supplement: Supplementary file 2 — Reporting Summary [file 41467_2025_63776_MOESM2_ESM.pdf]

nature portfolio

Corresponding author(s): Chun Cheng

Solar Cells Reporting Summary

Nature Portfolio wishes to improve the reproducibility of the work that we publish. This form is intended for publication with all accepted papers reporting the characterization of photovoltaic devices and provides structure for consistency and transparency in reporting. Some list items might not apply to an individual manuscript, but all fields must be completed for clarity.

For further information on Nature Research policies, including our data availability policy, see Authors & Referees.

Experimental design

Please check the following details are reported in the manuscript, and provide a brief description or explanation where applicable.

1. Dimensions

Item	Check	Description / explanation
Area of the tested solar cells	[x] Yes	The certificated aperture area is 0.085 cm2.
	[ ] No	*Explain why this information is not reported/not relevant.*
Method used to determine the device area	[x] Yes	The area is certified by an accredited laboratory through microscope.
	[ ] No	*Explain why this information is not reported/not relevant.*

2. Current-voltage characterization

Item	Check	Description / explanation
Current density-voltage (J-V) plots in both forward and backward direction	[ ] Yes	The hysteresis in J-V curves is not directly related to the batch characteristics of the devices.
	[x] No	
Voltage scan conditions	[x] Yes	the voltage step of 0.02 V and the delay time of 10 ms
	[ ] No	*Explain why this information is not reported/not relevant.*
Test environment	[x] Yes	room temperature; N2; glove box
	[ ] No	*Explain why this information is not reported/not relevant.*
Protocol for preconditioning of the device before its characterization	[ ] Yes	*Provide a description of the protocol.*
	[x] No	No preconditioning protocol was used.
Stability of the J-V characteristic	[x] Yes	The stabilized power output was provided in Fig. 4(f).
	[ ] No	*Explain why this information is not reported/not relevant.*

3. Hysteresis or any other unusual behaviour

Item	Check	Description / explanation
Description of the unusual behaviour observed during the characterization	[ ] Yes	*Provide a description of hysteresis or any other unusual behaviour observed during the characterization.*
	[x] No	No abnormal phenomena were observed during the characterization process.
Related experimental data	[ ] Yes	*Provide a description of the related experimental data.*
	[x] No	There are no abnormal behaviors, and the relevant data is relatively unremarkable.

4. Efficiency

Item	Check	Description / explanation
External quantum efficiency (EQE) or incident photons to current efficiency (IPCE)	[x] Yes	The EQE spectra were provided in Fig. 4(e).
	[ ] No	*Explain why this information is not reported/not relevant.*
A comparison between the integrated response under the standard reference spectrum and the response measure under the simulator	[x] Yes	Less than 5%.
	[ ] No	*Explain why this information is not reported/not relevant.*

For tandem solar cells, the bias illumination and bias voltage used for each subcell	☐ Yes ☒ No	*Provide a description of the measurement conditions.* This study did not involve tandem devices.<

5. Calibration

Light source and reference cell or sensor used for the characterization	☒ Yes ☐ No	Enlitech SS-X50 solar simulator with a 300 W Xenon lamp (Class AAA) was used for lab measurement. AAA steady state solar simulator (YSS-T155-2M) was used for certification. *Explain why this information is not reported/not relevant.*
Confirmation that the reference cell was calibrated and certified	☒ Yes ☐ No	Mono-Si, WPVS, calibrated by NREL (Certificate No. ISO 2075). *Explain why this information is not reported/not relevant.*
Calculation of spectral mismatch between the reference cell and the devices under test	☒ Yes ☐ No	The spectral mismatch factor was calculated based on IEC 60904-7 and I-V correction according to IEC 60891. *Explain why this information is not reported/not relevant.*

6. Mask/aperture

Size of the mask/aperture used during testing	☒ Yes ☐ No	Certified aperture area is 0.085 cm2. *Explain why this information is not reported/not relevant.*
Variation of the measured short-circuit current density with the mask/aperture area	☐ Yes ☒ No	*Report the difference in the short-circuit current density values measured with the mask and aperture area.* No significant variations were observed.

7. Performance certification

Identity of the independent certification laboratory that confirmed the photovoltaic performance	☐ Yes ☒ No	*Identify the independent certification laboratory.* Our work did not take any performance certification.
A copy of any certificate(s)	☐ Yes ☒ No	*Certificate copies should be provided in the Supplementary information. Please state the supplementary item number.* Our work does not claim record-certified performance.

8. Statistics

Number of solar cells tested	☒ Yes ☐ No	20 devices under each condition are shown in Fig. 4d. *Explain why this information is not reported/not relevant.*
Statistical analysis of the device performance	☒ Yes ☐ No	The statistical analysis was provided in Fig. 4(d). *Explain why this information is not reported/not relevant.*

9. Long-term stability analysis

Type of analysis, bias conditions and environmental conditions	☒ Yes ☐ No	Based on the International Summit on Organic Photovoltaic Stability (ISOS) protocols, ISOS-L-3 assessments at maximum power point tracking for 1830 h (Fig. 4g). *Explain why this information is not reported/not relevant.*
